# Knowledge of semantic categories in normal aged: Influence of education

**DOI:** 10.1590/s1980-57642008dn10200009

**Published:** 2007

**Authors:** Karla Shimura Barea, Leticia Lessa Mansur

**Affiliations:** 1 Course of Gerontology. Geriatric Service - Department of Clinics. Faculty of Medical Sciences of University of São Paulo.; 2 Lecturer of Speech Therapy Course. Department of Physiotherapy, Speech Therapy and Occupational Therapy. Faculty of Medical Sciences of University of São Paulo. Researcher of Group of Cognitive and Behavioral Neurology. Division of Neurology. Hospital of Clinics - Faculty of Medical Sciences of University of São Paulo.

**Keywords:** memory semantics, aged, schooling, language

## Abstract

Semantic memory seems to resist the effects of time, remaining stable even in more advanced ages.

**Objective:**

To verify the effect of schooling level on semantic knowledge (non-living items) in normal aged.

**Method:**

48 aged individuals were divided into three groups (based on schooling) and evaluated. Three tests were applied: verbal fluency, naming and figure classification.

**Results:**

We verified that the group with greater schooling (>8 years) differed to the illiterate and low schooling groups in most of the tasks, evoking more items in verbal fluency, correctly naming more items and presenting a greater number of “formal categories”.

**Discussion:**

In the verbal fluency test, this difference could be explained by the types of strategies used by the individuals to recall words. In relation to the naming test, the effect could be attributed to limitation in the vocabulary and cognitive processing skills needed to search for semantic attributes of the figures. In categorization, this type of classification is dependent on scholastic learning.

**Conclusion:**

We concluded that both illiterate elderly and those having a low schooling level, presented poorer performance in semantic memory tests compared to the aged with a higher level of schooling. The similar behavior evidenced between illiterate and low schooling groups is intriguing. It remains unclear whether the low schooling group behaved like the illiterates or vice-a-versa. This unanswered question remains the subject of future studies.

## Abstract (pt)

A memória semântica parece resistir aos efeitos do tempo e mantém estabilidade mesmo em idades mais avançadas.

**Objetivo:**

Verificar o efeito de escolaridade no conhecimento semântico de categorias inanimadas em idosos normais.

**Método:**

48 indivíduos idosos foram divididos em três grupos baseados no nível de escolaridade e avaliados. Três testes foram aplicados: fluência verbal, nomeação e classificação de figuras.

**Resultados:**

Houve diferença significativa no desempenho dos grupos, nos três testes: fluência verbal, nomeação e categorização. Discussão: A diferença entre os grupos no teste de fluência verbal pode ser explicada pela disponibilidade de maior número de estratégias eficientes no grupo escolarizado, para resgatar os itens. Em relação ao teste de nomeação, a desvantagem do grupo menos escolarizado pode ser atribuída à restrição de vocabulário e habilidades para identificar os atributos semânticos dos itens, mais limitada nos indivíduos com menor escolaridade. Na categorização de figuras, o grupo com maior escolaridade apresentou maior número de “categorias formais”, cujo conhecimento depende do aprendizado escolar.

**Conclusão:**

Os idosos com restrito acesso à instrução formal fornecida pela escola, apresentam desempenho pior nos testes de memória semântica, quando comparados com idosos escolarizados. O comportamento similar dos grupos analfabeto e pouco escolarizado é intrigante. Não sabemos se os indivíduos analfabetos se comportaram como os pouco escolarizados ou se os indivíduos pouco escolarizados se aproximaram dos analfabetos. Esta questão não respondida permanece como motivação para futuros estudos.

Memory in humans can be defined as the cognitive capacity allowing storage and retrieval of stimuli, being an essential element in the learning process.^1^

For the purposes of study, memory can be divided into short-term (system to store information for brief time intervals) and long-term (storing for long periods of time). Long-term memory has two storage components, namely, explicit and implicit memory. Explicit memory is a type of information storage easily accessed by the conscious mind (e.g. cognitive processes, including language).^2^

Explicit memory can also be divided according to the nature of the content, into episodic and semantic sub-systems.^2^

Episodic memory holds information on personal experiences in time and space while semantic memory permits the individual to codify and store knowledge about objects, facts and concepts^3^, such as words and their meanings. It is responsible for the maintenance of information, descriptions of events, vocabulary and meaning during one’s life.^4^

In traditional Quillian theory of semantic memory organization^5^, the storing of the concepts occurs in terms of definition of attributes or characteristics. The concepts are represented by hierarchies of knots of inter-related attributes. There are ordinate knots (e.g. mammals), supra-ordinate (e.g. animals) and subordinates (e.g. dogs). Currently, it is accepted that organization is dynamic and that a connections networks exists, activated according to the experience.

Semantic memory seems to be resistant to the effects of ageing and maintains this stability even at more advanced ages.^4^ In a project involving the naming of 85 figures in the young and elderly, it was observed that individuals older than 70 years represented a significantly greater number of errors than the young.^6^ Elderly individuals with a higher schooling level presented, apparently, a slower deterioration in comparison to individuals with lesser schooling.^7^ In other words, this population was able to maintain their cognitive and language skills for a longer period. In a similar study, there was a positive correlation found between the level of schooling and semantic knowledge.^8^

Studies on cognitive alterations have indicated that semantic memory also presented loss in Alzheimer’s dementia, albeit in a milder manner than episodic.^9^ This behavior is diverse in the semantic dementias.^3^

Semantic alterations do not necessarily mean deterioration in semantic memory. This type of memory suffers from the influence of other factors, such as difficulty in search strategies, difficulty in lexical access, or even, in the decrease of semantic memory activity.^10^

Warrington’s *bottom-up theory* states the hypothesis that individuals undergoing a process of dementia present a loss in access to attributes of categories and concepts, principally in the more specific areas, with relative maintenance of generic areas. Nonetheless, it was observed that performance of the analyzed groups did not correspond with the above-mentioned theory.^11^

There is still no consensus on the influences of the ageing process and neurological lesions on semantic memory.

It is known that schooling has an influence in the performance of semantic tasks, such as naming and verbal fluency.^12,13^

In the specialized literature, various protocols can be found evaluating the functioning of memory. Visual stimuli for the categorization process present answers given more quickly for non-related categories yet slower for conditions of similarity, this difference being greater than that occurring with verbal stimuli.^14^

The use of figures as stimuli facilitates the study of semantic memory in individuals with a low level of schooling or who are illiterate.

Thus, understanding categorization skills and the hierarchy of concepts in the healthy aged, along with possible effects of socio-cultural variables, is important to measure alterations in pathological conditions.

The observation of elderly Brazilians with low schooling in semantic memory tasks related to inanimate knowledge can contribute to the future adaptation of material to be used on the Brazilian population.

The objective of this study was to verify the effect of schooling level on semantic knowledge of non-living categories in the healthy aged.

## Methods

### Casuistic

The sample consisted of 48 elderly individuals, aged between 60 and 85 years (mean 66.9 years), of both genders (32 women and 15 men). The educational level of the sample varied from zero to 19 years. The participants were divided into three groups, according to their formal education: group A (illiterate), group B (1-8 years of schooling) and group C (more than 8 years of schooling). Groups A, B and C included 6,23 and 19 participants, respectively. The mean age of group A was 74.17 years; group B, 67.04 years and group C, 64.53 years.

The data was collected at a Specialized Out-Patient Center for the Aged in the Public System located in the outlying district of the city of São Paulo.

Only those aged fulfilling the criteria of normalcy as established by the Mayo Older American Normative Studies (MOANS):^15^ absence of an active psychiatric or neurological disease; absence of complaint of cognitive difficulty during the interview; absence of psychotropic medication in quantities that could compromise cognition or suggest neuro-psychiatric disturbances; independent living status in the community; while history of progressive disturbances (e.g. alcoholism) with potential to affect cognition are not automatically excluded, provided the disturbances are not active and had been resolved without any apparent cognitive sequels. Chronic medical illnesses were not criteria for exclusion, given the condition had not been reported by the physician as being responsible for the cognitive compromise. Individuals with some degree of visual or auditory deficiency, not compensated with correctives devices, were not included.

Three elderly were excluded from the sample because they were using drugs with potential action on the central nervous system.

The study was approved by the Research Ethics Committee of Hospital das Clínicas – FMUSP and all participants signed the Informed Consent

### Procedures

Three tests were utilized to evaluate semantic memory: verbal fluency test, naming and free categorization of figures. 80 figures were used in the non-living categories of the Florida Semantic Battery.^16^ This consists of stimuli in black-and-white, with representation corresponding to the prototypical image of the item. In other words, the stimuli are marked by a strongly corresponding appearance. The same stimuli were used in naming and categorization tasks.

The figures were divided into eight distinct categories: clothing, office-items, means of transportation, kitchen utensils, tools, musical instruments, personal items and furniture. The categories were pre-established by the test of origin and have been called “formal categories” in order to facilitate data analysis.

In verbal fluency, the “supermarket items” category was used, given we had decided to analyze the knowledge of non-living things.

The tests were applied individually. For those that needed corrective lenses in order to see, the test was applied only while these were being used.

### Tests

*Verbal fluency* – Participants were instructed to evoke the greatest number of supermarket items possible in one minute. The total number of elements evoked was calculated, excluding repetitions, gender variations, number and degree, as well as synonyms for the same item.

*Naming* – The figures were presented randomly one at a time, and the individual was asked to name them. Both right and wrong answers by the individual were considered. The errors were classified as: no-answer (does not remember what it is called, or does not know), semantics (semantically related to the target) and non-pertinent (visual error or without classification). Responses were deemed correct when the label for the stimuli was given.

*Categorization* – The participants were instructed to group all the figures into free-form categories. This procedure was performed without a fixed time limit or pre-existing categories. The groups presented by the subject were analyzed as “formal categories” (mentioned above) or by sub-categories.

For statistical analysis, we used Mann-Whitney, Spearman’s Correlation, and Kruskal-Wallis where the level of significance considered was 0.05 (5%). The non-parametric tests were chosen because the data did not obey normal distribution.

## Results

The educational level of the participants was the reference used to constitute the three groups, but there was an interaction factor of age in group A: this group of illiterates were older than the participants from the other groups: group A X B, p=0.021; AXC, p=0.001. Groups B and C however, were similar in terms of age of participants. The performance of group A was influenced by age and schooling.

No influence of gender on verbal fluency, naming and categorization tasks (Table 1) was observed in our sample.

**Table 1:** Performance by gender in verbal fluency, naming (correct answers) and categorization tasks.

	Verbal fluency			Naming			Formal categories	
	Fem	Male		Fem	Male		Fem	Male
Genders	N=32	N=16		N=32	N=16		N=32	N=16
Mean value	17.38	16.94		60.66	61.63		5.38	5.81
Median	18.00	18.00		60.50	65.00		6.00	6.00
Standard deviation	5.21	3.28		10.33	12.48		1.95	2.07
p-value	1.000			0.710			0.437	

Considering the three groups, we analyzed verbal fluency, naming and categorization tasks to observe the influence of educational level on performance.

In verbal fluency, we noted significant difference in the behavior of groups A, B and C: group C was distinct from groups A and B in that highly educated participants evoked a higher number of items in this task.

In the naming task (Table 3), we verified significant difference in the number of pictures named correctly when the three groups were compared. The means of correct answers were: group A=60.0%, group B=70.7% and group C=88.1%,group C differing from B and C.

**Table 3:** Comparison of correct naming and types of errors in schooling groups.

					p-value			
				Standard	(Groups	Group	Group	Group
Naming and groups		Mean	Median	deviation	AxBxC)	AxB	BxC	AxC
Correct answers	A (N=6)	48.0	49.5	4.2				
	B (N=23)	56.5	55.0	9.1	<0.001*	0.066	0.002*	0.002*
	C (N=19)	70.5	10.0	5.7				
No answer	A (N=6)	11.0	8.5	6.4				
	B (N=23)	6.0	5.0	4.3	<0.001*	0.339	<0.001*	<0.001*
	C (N=19)	2.3	1.0	2.9				
Semantic errors	A (N=6)	14.0	13.5	3.1				
	B (N=23)	12.2	13.0	3.7	<0.001*	0.290	<0.001*	<0.001*
	C (N=19)	6.2	6.0	3.3				
Errors totally unrelated	A (N=6)	7.0	7.0	1.1				
with the target	B (N=23)	5.4	6.0	4.0	<0.001*	0.049*	<0.001*	<0.001*
	C (N=19)	1.0	0.0	1.1				
Total errors	A (N=6)	32.0	30.5	4.2				
	B (N=23)	23.5	25.0	9.1	<0.001*	0.049*	<0.001*	<0.001*
	C (N=19)	9.5	10.0	5.7				

* significant value.

The type of errors presented (Table 3) differed among the three groups. Groups A and B performed similarly while group C (high educational level) differed. In other words, there was a strong relationship between low number of errors and high schooling. The exception was the comparison between the numbers of non-pertinent errors across the three groups: group A (low educational level) differed from group B (1-8 years of schooling). In the other types of errors, group A behaved in a similar manner to group B, although the participants in this group were older and less educated than the latter.

Similar behavior was also noted in the high correlation between verbal fluency and naming tasks (Table 4) where there was positive strong correlation between performance in verbal fluency and naming in groups A and B.

**Table 4:** Correlation of performance of schooling groups in verbal fluency and naming tasks.

Verbal fluency		
Naming	Correlation	p-value
Group A	88.20%	0.020*
Group B	47.80%	0.021*
Group C	-16.10%	0.509

* significant value.

In the categorization task, we used the pre-established categories of the Florida Semantic Battery as a reference for “correct” categorization. Table 5 shows a significant difference among the groups in terms of formal categories and sub-categories. In the comparison of the three groups, we again observed that performance of group C was different from A and B. There was positive correlation between years of education and the capacity to organize categories and sub-categories.

**Table 5:** Number of formal categories and sub-categories performed by schooling groups.

	Formal categories				Sub-categories		
Categorisation	Group A N=6	Group B N=23	Group C N=19		Group A N=6	Group B N=23	Group C N=19
Mean	3.83	4.96	6.74		7.17	5.52	2.63
Median	4.00	5.00	7.00		7.00	5.00	2.00
Standard deviation	1.33	1.82	1.66		2.79	3.96	3.47
p-value		0.001*				0.004*	

Note: comparison of formal categories performed by Groups (p value): AxB=0.112; BxC=0.001*; AxC=0.003*. Comparison of formal sub-categories performed by Groups (p value): AxB=0.203; BxC=0.007*; AxC=0.006*.

* statistically significant.

## Discussion

The rationale for this study was to analyze the performance in semantic memory of aged Brazilians. It is widely-known that semantic memory is relatively spared in the ageing process^16,17^ and an alteration of this type of memory frequently results in cognitive deterioration. There are a number of studies on semantic processing and degenerative or dementia illnesses.^9,11,22-24,27^ However, few have been performed with the aim of verifying performance in individuals over 60 years of age and having a low level of schooling.

Our results do not confirm the influence of gender, while the literature favors women in semantic memory tests, particularly in phonemic and semantic verbal fluency.^25,26^

In analyzing the verbal fluency test for inanimate objects (supermarket items) we verified a positive correlation between years of schooling and the number of items evoked in one minute. This result is in agreement with the results of Porto^18^ in the Portuguese language but differs for the verbal fluency test for foods and animals in a Portuguese language population, where these authors observed a significant difference only in the animals category (the groups presented similar evocations for supermarket items).^19^

In the Brazilian population, a significant difference was found between the two groups of schooling levels (low and high schooling levels) in the verbal fluency of animals.^13^ This study also demonstrated that age seems not to interfere in the performance of the test. The authors concluded that there is a strong indication that schooling influences performance of the test in question.

The objective of the verbal fluency test is to evaluate executive functions and semantic memory. It is well known that the test performed with categories is more sensitive in detecting alterations in semantic memory than when realized with letters (e.g. *“Say the greatest number of words that you can beginning with the letter S”*). Moreover, it is easier to apply the categories test with illiterate individuals, than using letters which requires a minimum of schooling.

Furthermore, verbal fluency depends on skills in the use of search strategies and the organization of the semantic terms necessary for word retrieval. For this skill, it is necessary to access the semantic domain and select which should be evoked from the sub-categories.^18^

The result obtained can explain, therefore, the improved evocation of the items of the category by the individuals with more schooling, in other words, with greater organization of the strategies used for word retrieval. However, a study testing by items and their categories was not undertaken here.

In the naming test we observed significant difference in the behavior of the three groups, where group C again differed to group A and B. Schooling positively influenced the number of items named correctly.

In the analysis of types of errors, we observed that the groups A and B presented similar performance, and differed to C, except in the occurrence of errors of the “non-pertinent” type, in which the three groups differed. This result indicates that fewer errors were related to high degree of education. In our sample, “semantic” errors predominated and the answers were related to the target, indicating that most participants identified the visual characteristics of the stimuli.

The effect of schooling was verified in the Boston Naming Test, in elderly aged more than 80 years-old and with low educational level.^20^ This is in line with other studies of Brazilian elderly.^8^

The greater number of correct answers in the naming test can be attributed to the broader and more accurate vocabulary used by subjects with a high level of schooling. Additionally, these individuals possess greater skills in searching for the semantic attributes of the figures. The degree of familiarity with the presented figures must also be taken into consideration, principally in relation to the group with a low level of schooling. In this test, we hypothesized that the illiterate elderly would present similar retrieval processing of elderly with 1-8 years of schooling, due to the demand of task in employing a precise lexical label.

In studies on adult Brazilian university students, there were no significant errors in naming. The naming in this study did not present a relationship between familiarity and visual complexity as seen in other studies.^21^

In analyzing a possible correlation of performance of the groups in the naming and verbal fluency tasks, we observed that there was a similar positive correlation in performance of groups A and B, where higher number of correct items named corresponded with higher number of items retrieved in verbal fluency. To our knowledge, no studies of illiterate and low-educated elderly have been performed.

In the categorization test, we also observed differences among group C and the two other groups. Group C presented a greater number of formal categories when compared with the others. Once again, the difference occurred with the individuals who had more education. We did not explore the possible strategies employed by the individuals.

The sensorial/functional theory proposed by Warrington and Shallice and cited by Laiacona et al.,^22^ raises the hypothesis that the concepts are characterized by different sensorial or functional properties for the identification of a categorization type. The sensorial properties and semantic and visual knowledge should be crucial to the identification of the animate categories (living), and the functional properties would be related to the inanimate categories (non-living). According to this theory, the demential processes should initially affect the categories of living things.

This theory would explain the performance of group A and B in the categorization task, as, besides presenting a greater number of sub-categories, we also observed that the categories they formulated were associated to personal experience, through the cultural and functional practice of activities. Furthermore, these individuals presented fewer cognitive strategies supported by abstraction.

Additionally, “formal categories” can be considered as classifications learned during the literacy process and during continued education. The access to formal instruction, thus, leads to differences among the groups. Therefore, the positive relationship between low level of schooling and sub-categories can be explained by the functional and practical knowledge that these subjects make use of to form the categories. Nonetheless, it cannot be inferred that these strategies are less efficient for cognitive operations.

In reviews on semantic memory, there is a consensus that categories of animate beings presented a greater number of inter-related semantic properties, while categories of non-living entities presented a greater proportion of distinct and less-related properties. There are divergences in relation to the acceptance of the sensorial/functional theory as a basis to explain the pathological behaviors in AD.^23, 24^

Further research on this question is necessary to better understand the processing of this type of memory in elderly subjects with different degrees of formal education.

Additionally, there is a need for studies on the associative norms in Portuguese for semantic categories. In a study on association of words to categories, developed at the University of Brasilia, the author himself recognized the need to continue such studies into the cultural basis of semantic memory organization, with the aim of outlining norms for the semantic categories applicable to the Brazilian population.^28^

The present study has furthered knowledge on the performance of aged Brazilians in semantic memory tests, which has led us to conclude that the aged with a low level of schooling presented reduced performance in semantic memory tests compared to aged subjects with a higher level of schooling. Education above 8 years was associated to significant differences in the performance of elderly in semantic memory tests. It is important to highlight the similar behavior of illiterate and low educated (1-8 years) elderly observed. An intriguing question to be elucidated, in future studies, is whether illiterate elderly behave similarly to the 1-8 year - education group, or whether the low-schooling group behave similarly to the illiterates.

## Figures and Tables

**Table 2:** Performance of groups in verbal fluency.

Verbal fluency	Group A N=6	Group B N=23	Group C N=19
Mean	14.50	16.48	19.00
Median	12.50	17.00	20.00
Standard deviation	5.05	5.37	3.21
p-value	0.021*		

Comparison of groups (p value): AxB=0.212; BxC=0.021*; AxC=0.041*

* significant value.
